# Trabecular bone score in type 1 diabetes: a meta-analysis of cross-sectional studies

**DOI:** 10.1186/s13018-023-04289-0

**Published:** 2023-10-24

**Authors:** Runzhou Pan, Yan Zhang, Yongcai Zhao

**Affiliations:** Department of Endocrinology, Cangzhou Central Hospital, Cangzhou, Hebei Province China

**Keywords:** Type 1 diabetes, Trabecular bone score, Meta-analysis

## Abstract

**Background:**

Bone fragility is a recognized complication of type 1 diabetes (T1D). Thus, lower trabecular bone score (TBS) measurements in T1D patients can be predicted. However, the results of current studies on TBS in patients with T1D are inconsistent. In this context, the present study aimed to test the hypothesis that T1D is associated with lower TBS through a meta-analysis.

**Methods:**

An electronic search of the literature was conducted using PubMed, Embase and Web of science databases to identify studies related to TBS and T1D, supplemented by an additional manual check of the reference list of relevant original and review articles. All data was analyzed using a random effects model. Results were compared using standardized mean differences (SMD) and 95% confidence intervals (CI). *P* ≤ 0.05 was considered statistically significant. Review Manager 5.4 software and Stata 17.0 software were used for statistical analysis.

**Results:**

Seven cross-sectional studies involving 848 participants were included. TBS was lower in T1D patients than in healthy controls on random effects analysis, with no heterogeneity (SMD =  − 0.39, 95% CI [− 0.53, − 0.24], *P* < 0.001; I^2^ = 0%). In addition, by subgroup analysis, T1D patients were strongly associated with reduced TBS in different regions and age groups, and the results were independent of covariate adjustment.

**Conclusion:**

This study showed that TBS was lower in patients with T1D than in healthy individuals with normal blood glucose levels, suggesting that TBS may be a useful measure to assess fracture risk in T1D.

**Supplementary Information:**

The online version contains supplementary material available at 10.1186/s13018-023-04289-0.

## Introduction

Type 1 diabetes (T1D) is an autoimmune disease characterized by the loss of pancreatic beta cells that secrete insulin [1]. Improved insulin therapy has reduced life-threatening complications and increased longevity in T1D patients. However, T1D's damage to bone health needs more attention. Studies have confirmed that people with T1D generally have low bone mineral density (BMD) [2] and T1D is associated with an increased risk of osteoporotic fractures at any age and gender [3, 4]. However, the relatively small reduction in BMD does not fully explain the increased risk of fracture in patients with T1D, and the actual fracture rate largely exceeds the calculated risk of fracture based on BMD measurements, suggesting that T1D adversely affects bone quality [2].

Bone histomorphometry and quantitative computed tomography, the standard methods for evaluating bone quality, have limitations: bone biopsies are invasive, and quantitative computed tomography results in radiation exposure and high costs. The trabecular bone score (TBS) made up for the above shortcomings. The microstructure of the trabecular bone is an important component of bone quality, and TBS is a non-invasive tool to measure the trabecular bone, which can be obtained from spine BMD scans of the spine by determining the slope of the logarithmic transformation of the two-dimensional variation map associated with the gray level in dual energy X-ray absorptiometry (DXA) images [5]. Unlike BMD, which reflects bone mass, the TBS measurement, which reflects changes in trabecular composition or bone microarchitecture in the trabecular-rich lumbar spine, is positively correlated with standard 3D bone microstructural parameters such as junction density and trabecular number [6, 7]. Recent study has found that the trend of TBS is not consistent with BMD in different age groups, indicating that TBS reflects developmental differences in bone microstructure and bone minerals [8]. The risk of fracture depends on bone strength, including bone mass and bone quality. Because BMD can only be used to assess bone mass but not bone quality, BMD often underestimates the risk of fracture in the population. TBS can be used as an adjunct measure of BMD and as an assessment tool for the risk of osteoporotic fractures [9]. Higher TBS reflects higher fracture resistance in denser bones. Low TBS values were found to be associated with an increased risk of fragility fractures, regardless of BMD and age [10].

Bone fragility is a recognized complication of T1D and type 2 diabetes mellitus (T2D) [11]. The change in TBS in individuals with diabetes deserves attention as an indicator of bone quality. A recent meta-analysis confirmed that patients with T2D had lower TBS than healthy people [12]. Therefore, we can predict that, like in individuals with T2D, TBS measurements would be lower in individuals with T1D. However, the results of current studies on TBS in patients with T1D are not consistent, with some studies reporting lower TBS levels in T1D patients than in controls [13], while others found no difference [14]. In this context, this meta-analysis aimed to examine the hypothesis of T1D being associated with lower TBS by collecting peer-reviewed published evidence on the differences in TBS between T1D and healthy subjects.

## Methods

### Search strategy and study inclusion

An electronic search of the literature was conducted using PubMed, Embase and Web of science databases to identify studies related to TBS and T1D, supplemented by an additional manual check of the reference list of relevant original and review articles. The last retrieval time was on September 1, 2023. The initial search terms included “type 1 Diabetes” OR “Insulin-Dependent Diabetes Mellitus” OR “Juvenile-Onset Diabetes Mellitus” OR “Sudden-Onset Diabetes Mellitus” OR “Autoimmune Diabetes” OR “Brittle Diabetes Mellitus” OR “Ketosis-Prone Diabetes Mellitus” OR “Trabecular bone score” OR “TBS” OR “Osteoporosis” OR “Bone health”. The inclusion criteria were (a) original studies published in English journals reporting data on TBS and T1D; (b) observational studies; (c) TBS was evaluated using iNsight software in DEXA technology. Reviews, case reports, conference papers, or animal studies were excluded. Two reviewers independently appraised qualified articles according to the above criteria. Differences in opinion as to whether research should be included in the analysis have been resolved through discussion.

### Data extraction and synthesis

Data extraction was conducted independently by two researchers. For each study, we extracted data related to study characteristics and outcomes: author, journal, year of publication, study design, country, age, sex, number of participants, and TBS. All data are presented as means and SDs. Some studies report median and quartile spacing, and we estimate standard deviations using the methods described by Wan et al. [15]. When studies report unadjusted and adjusted means, we use adjusted means for meta-analysis. All data was analyzed using a random effects model. Results were compared using standardized mean differences (SMD) and 95% confidence intervals (CI). *P* ≤ 0.05 was considered statistically significant. Statistical heterogeneity was tested by I^2^. I^2^ lower than 50% was considered low heterogeneity, I^2^ between 50 and 75% was considered moderate heterogeneity, and I^2^ greater than 75% was considered significant heterogeneity. In addition, a sensitivity analysis was performed to test the robustness of the results.

We utilized the Newcastle–Ottawa Scale (NOS) to assess the quality of the included studies for quantitative analysis. The current NOS is only applicable to case–control and cohort studies. Therefore, we employed an adapted version of NOS to evaluate the quality of cross-sectional studies. Studies with a NOS score of ≥ 7 are considered to be of high quality [16] (see Additional file 1: Table S1).

Subgroup analyses were performed by region, age and whether the outcome was adjusted for covariates. Publication bias was analyzed by visual inspection of the funnel plot and the Egger test. Review Manager 5.4 software and Stata17.0 software were used for statistical analysis.

## Results

### Literature search

**Fig. 1:**
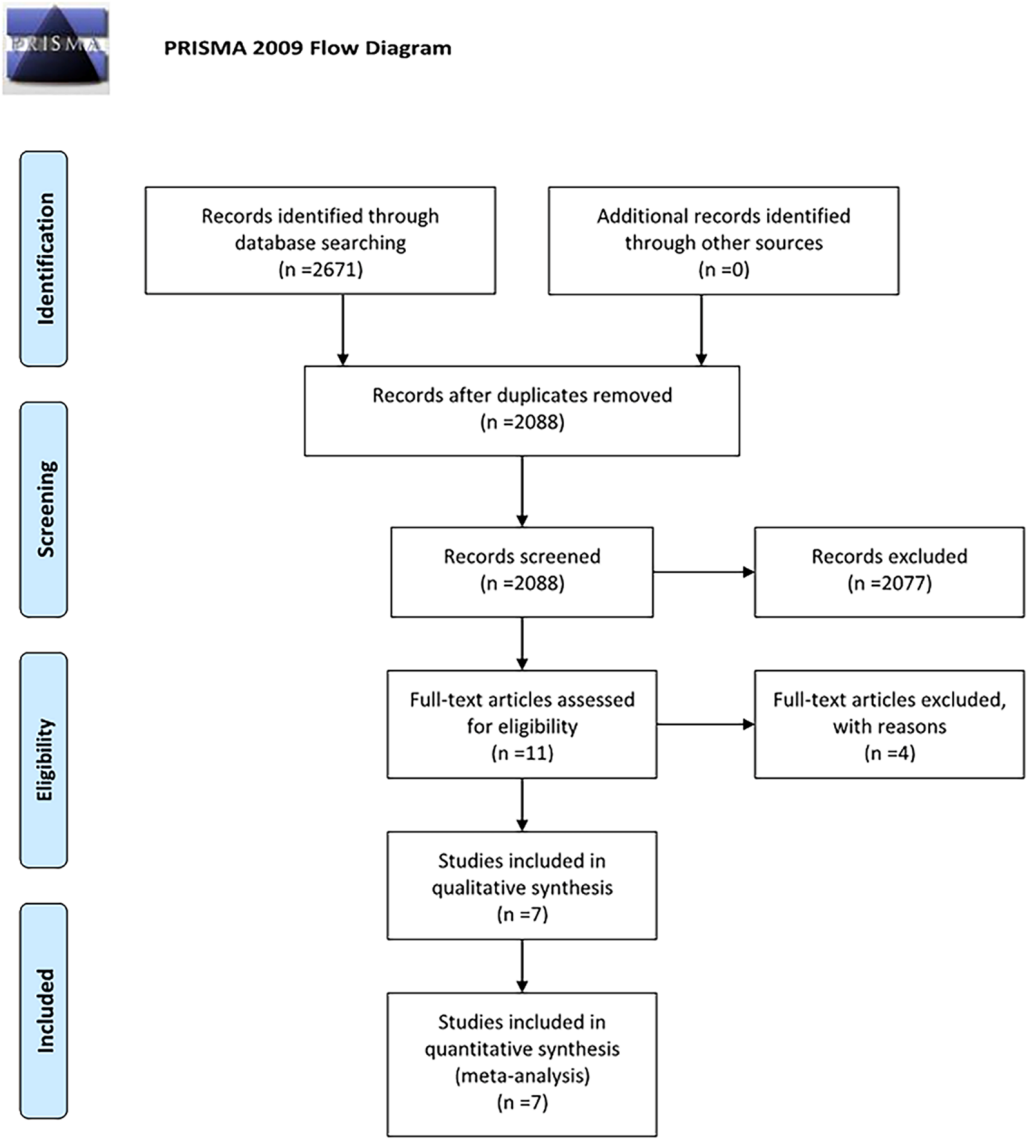
Study flow diagram. From: Moher D, Liberati A, Tetzlaff J, Altman DG, The PRISMSA Group (2009). Preferred reporting items for systematic reviews and meta analyses: The PRISMA statement. PLoS Med 6(7): e1000097. Doi:10.1371/journal.pmed1000097. For more information, visit www.primsa-statement.org

Through an initial search strategy, we identified 2671 studies (395 in PubMed, 661 in Embase, and 1342 in Web of Science). After eliminating repeated trials (n = 583), the remaining 2088 studies were screened by title and abstract, and a further 2077 studies were excluded, including reviews (n = 174), animal studies (n = 71), irrelevant studies (n = 1824) and conference studies (n = 8). Full text browsing was performed for the remaining 11 studies. Four studies were excluded for not reporting TBS data (n = 2), no distinction was made between diabetic types (n = 1) or having no control group (n = 1). Finally, seven studies were included [13, 14, 17–21] (see Fig. 1).

### Characteristics of studies

Seven cross-sectional studies involving 848 participants were included. 1 study was conducted on Asian populations [21], 2 studies were on European [14, 18], 3 studies were on North American [13, 19, 20], and 1 study was on South American [17]. One study was conducted on men [18], 5 studies included men and women [13, 14, 17, 19, 20] and one study was conducted on children [21]. Dual energy x-ray absorptiometry manufacturers (DXA) included Hologic inc. and GE Lunar. Based on gray level analysis of DXA images, TBS was calculated as the average of each measured value of the vertebral body (see Table 1).

**Table 1:** Characteristics of individual studies relating TBS to T1D

References	Year	Countries	Study subjects	TBS software version	Adjustment for covariates
Shah VN	2018	USA	47 T1D (23 females/24 males) and 47 controls (27 females/20 males) aged > 18 years	Hologic inc	Age, BMI
Neumann T	2015	Germany	128 T1D (65 females/63 males) and 77 controls (39 females/39 males) aged > 18 years	GE Lunar	No adjustment
Carvalho AL	2018	Brazil	23 T1D (10 females/13 males) and 24 controls (11 females/13 males) aged 18–70 years	Hologic inc	No adjustment
Syversen U	2021	Norway	33 men with T1DM aged 20–62 years and 28 controls aged 23–63 years	Hologic inc	No adjustment
Thangavelu T	2020	USA	48 T1D (27 females/21 males) and 75 controls (43 females/32 males) aged 19–50 years	Hologic Inc	No adjustment
Coll JC	2022	Canada	127 T1D (70 females/57 males) and 65 controls (35 females/30 males) aged ≥ 20 years	Hologic inc	BMI
Wagh A	2021	India	137 children with T1DM and 68 children controls	GE Lunar	No adjustment

T1D, type 1 diabetes; TBS, trabecular bone score; BMI, body mass index

### TBS and T1D

**Fig. 2:**
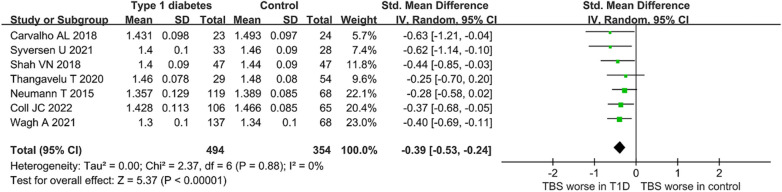
Forest plot of the difference in TBS between T1D and healthy controls for all studies

In general, TBS was lower in patients with T1D than in healthy controls in random effects analysis, with no heterogeneity (SMD =  − 0.39, 95% CI [− 0.53, − 0.24], *P* < 0.001; I^2^ = 0%) (see Fig. 2). Furthermore, the outlier study was not found by sensitivity analysis. Therefore, the robustness of the combined results of this meta-analysis was confirmed.

**Fig. 3:**
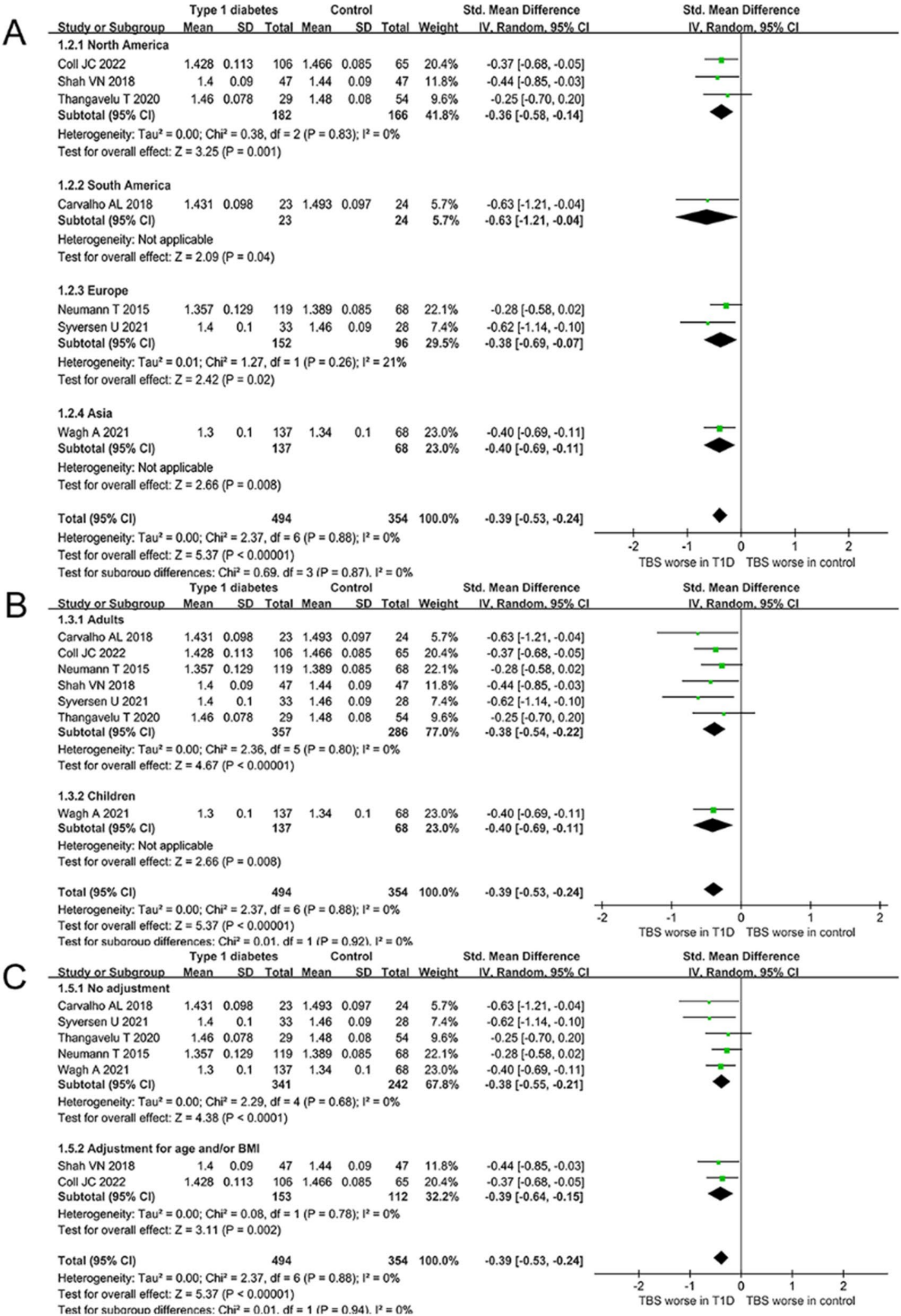
Subgroup analyses of the difference in TBS between T1D and healthy controls. **A** Subgroup analysis according to the region. **B** Subgroup analysis according to the age. **C** Subgroup analysis according to the adjustment of age and/or BMI

Further subgroup analyzes were performed to assess the effect of grouping factors on the results. First, we found a correlation between T1D and decreased TBS in Europe, Asia, North America, and South America (North America: SMD =  − 0.36, 95% CI [− 0.58, − 0.04], *P* = 0.001, I^2^ = 0%; South America: SMD =  − 0.63, 95% CI [− 1.21, − 0.04], *P* = 0.04, I^2^ = 0%; Europe: SMD =  − 0.38, 95% CI [− 0.69, − 0.07], *P* = 0.02, I^2^ = 0%; Asia: SMD =  − 0.40, 95% CI [− 0.69, − 0.11], *P* = 0.008) (see Fig. 3A). Second, T1D was associated with a decrease in TBS in both adults and children (Adults: SMD =  − 0.38, 95% CI [− 0.54, − 0.22], *P* < 0.001, I^2^ = 0%; Children: SMD =  − 0.40, 95% CI [− 0.69, − 0.11, *P* = 0.008) (see Fig. 3B). Finally, T1D was associated with decreased TBS regardless of whether TBS outcomes were adjusted for age and/or body mass index (BMI) (No adjustment: SMD =  − 0.38, 95% CI [− 0.55, − 0.21], *P* < 0.001, I^2^ = 0%; Adjustment for age and/or BMI: SMD =  − 0.39, 95% CI [− 0.64, − 0.15], *P* = 0.002, I^2^ = 0%) (see Fig. 3C).

### Publication bias

**Fig. 4:**
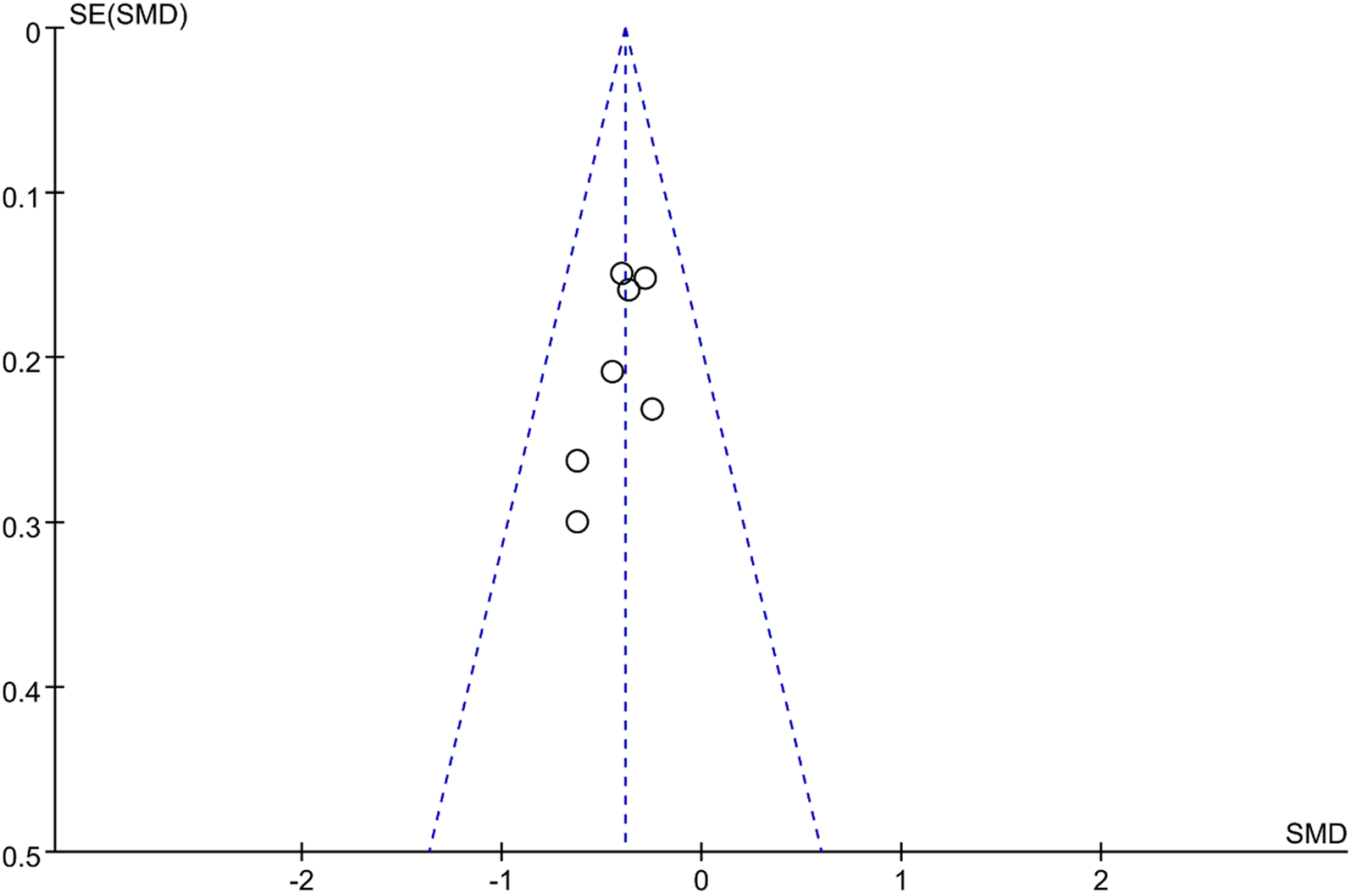
Funnel plots for the publication bias underlying the meta-analysis of the association in TBS between T1D and healthy controls

Visual inspection of the funnel plot is symmetric, indicating a low risk of publication bias (see Fig. 4). Egger's regression test also suggested a low risk of publication bias (*P* = 0.116).

## Discussion

Bone strength consists of bone mass and bone quality. BMD alone reflects only bone mass and does not fully reflect bone microstructure, and there is considerable overlap between BMD in fractured and unfractured patients [22]. TBS is a texture parameter related to bone microstructure, and it provides skeletal information that standard bone density measurements cannot obtain [23]. It measures the variation in grayscale texture from one pixel to the next in a two-dimensional image [6]. TBS has the potential to identify the differences in three-dimensional microstructure between two-dimensional DXA measurements with similar bone mineral density levels [6, 24]. Both in vitro and clinical studies have consistently found strong positive correlations between Trabecular Bone Score (TBS) and the ratio of bone volume (BV) to tissue volume (TV), trabecular number, trabecular connectivity, and trabecular hardness. Conversely, TBS has been found to have negative correlations with trabecular spacing, structural model index, and measurements of trabecular rods and plates [7, 25, 26], confirming the role of TBS in assessing bone quality. Under the premise of the same BMD, higher TBS values are associated with stronger fracture-resistant microstructure, while lower TBS values are associated with weaker fracture-prone microstructure. TBS provides an independent prediction of fragility fractures, regardless of BMD [27, 28].

There is a significant discrepancy between the fracture risk calculated based on BMD measurements and the actual observed fracture rates in both T1D and T2D patients [2]. Therefore, abnormal bone microstructure and bone quality may be another important factor contributing to the increased risk of diabetes-related fractures. Studies using Quantitative Computed Tomography or magnetic resonance imaging to assess bone microstructure have found that in patients with T1D, there is an increase in trabecular separation and a decrease in trabecular number, volume, and thickness [29, 30]. Previous research has established that TBS is an independent predictor of diabetes-related fractures, regardless of BMD [31, 32]. A recent meta-analysis has confirmed that T2DM is associated with lower TBS [12]. Given the assumption mentioned above, it is expected that TBS levels would be lower in patients with T1D compared to non-diabetic individuals. Consistent with our expectations, the findings from our meta-analysis provide additional support for the hypothesis that T1D is linked to decreased TBS levels. The mechanisms underlying the decrease in bone mass, increase in bone fragility, and elevated risk of fractures in patients with T1D are multifaceted: (1) The accumulation of advanced glycation end products (AGEs): Elevated glucose levels in diabetic patients contribute to the accumulation of advanced glycation end products (AGEs) in the organic bone matrix. The cross-linking of these AGEs results in increased fragility, loss of toughness, and reduced prefracture deformability of the bone [33]. (2) Low bone turnover and elevated sclerostin levels: Diabetic patients exhibit reduced bone turnover levels, characterized by decreased levels of bone resorption markers, including collagen C-terminal cross-linking, as well as bone formation markers such as osteocalcin and Procollagen type I N-terminal propeptide. These alterations contribute to heightened bone fragility [34]. In addition, increased expression of sclerostin, a major inhibitor of bone formation, has been demonstrated [35]. Increased sclerostin levels in diabetic patients are associated with decreased levels of bone formation markers such as β-catenin91, further suggesting that sclerostin inhibits bone turnover in the diabetic state [36]. (3) Excess bone marrow fat: Long-term hyperglycemia can activate peroxisome proliferator-activated receptor γ to promote the differentiation of bone marrow mesenchymal stem cells into adipocytes while reducing their differentiation into osteoblasts. Additionally, bone marrow adipocytes release free fatty acids, which generate reactive oxygen species that hinder osteoblast proliferation and function while inducing osteoblast apoptosis [37]. (4) Insulin, growth hormone (GH), and insulin-like growth factor-1 (IGF-1) deficiency: Both osteoblasts and osteoclasts express insulin receptors. In vitro and in vivo studies have demonstrated that insulin promotes bone formation [38, 39]. GH and IGF-1 play crucial roles in skeletal homeostasis and have significant implications for bone mass maintenance [40]. Insulin deficiency is associated with growth hormone resistance, and the resultant decrease in IGF-1 due to insulin deficiency in patients with T1D may contribute to skeletal abnormalities [30]. (5) Mineral metabolism disorders: T1D patients commonly experience disruptions in calcium, phosphorus, and magnesium metabolism, as well as vitamin D deficiency, which can impair the mineralization process [41]. (6) Inflammation and autoimmune factors: Patients with T1D exhibit autoimmune dysfunction, it is commonly associated with increased levels of IL-1, IL-6, and TNF-α t as well as decreased levels of the anti-inflammatory cytokine IL-10. These changes in factors result in decreased bone formation and increased bone resorption [42, 43]. (7) Loss of incretin action: The expression of GLP-1 receptors has been observed in bone marrow stromal cells and immature osteoblasts. GLP-1 has been demonstrated to stimulate the proliferation of mesenchymal stem cells and inhibit their differentiation into adipocytes [42]. Patients with diabetes have reduced incretin effects and impaired postprandial GLP-1 production [44] (8) Increased risk of falls: The use of insulin in diabetes treatment is associated with an elevated risk of falls due to several factors. This includes the severity of the disease, the presence of long-term conditions that can impair vision, peripheral neuropathy, decreased muscle function, chronic gait and/or balance disorders. These factors collectively contribute to an increased risk of falls, which in turn heightens the risk of fractures [42]. However, it is currently unclear which skeletal characteristics in T1D affect TBS. Therefore, further research is needed to explore the specific mechanisms underlying T1D-induced TBS decline.

The effect size we observed in this analysis was modest, with a difference of − 0.39 standard deviations in TBS between patients with T1D and non-diabetic individuals. For every standard deviation reduction in TBS, fracture risk increased by about 1.4 times [45]. Thus, it can be inferred from the results that TBS was reduced by approximately 0.40 standard deviations and the risk of fracture increased by 16% in T1D patients. Previous studies have confirmed that patients with T1D have an increased risk of general fracture compared with age—and sex-matched controls, with pooled relative risks ranging from 1.88 to 3.16 [46–48], as well as an increased risk of hip fracture and lumbar fracture, with pooled relative risks ranging from 3.78 to 6.30 and 2.88, respectively [3]. In addition, the risk of fracture was also increased in T1D compared to T2D, with a general relative risk of fracture of 1.24 and hip fracture of 3.43 [49]. Therefore, similar to the lower decrease in BMD, the decrease in TBS cannot fully explain the increased risk in patients with T1D. In addition to decreased bone mass and bone quality, age of onset, chronic complications, and poor blood glucose control are also risk factors for fracture in T1D [3, 50], indicating that the influence of T1D on fracture risk is multifactorial.

To further explore the effect of different population and study characteristics on the results, we performed subgroup analyses. First, T1D was strongly associated with decreased TBS regardless of region. However, TBS decreased to a greater extent in Asian and South American patients than in European and North American patients. This is similar to the most recent conclusions regarding the relationship between children with T1D and BMD: BMD was lower in children with T1D in South America and Asia, but there was no significant decrease in children with T1D in North America and Europe [51]. Previous studies have found that compared to Caucasians, Asians have a lower BMD, smaller bone size, smaller trabecular size, wider septal size, lower trabecular stiffness,, whole bone stiffness and failure load, which may be related to their lower height and weight [52]. Because Asian children had significantly lower physical activity and calcium intake than white children, Asian children had significantly lower BMD at bone sites than white children [53]. Therefore, it is necessary to promote an active lifestyle in different ethnic groups especially in Asian and South American T1D patients to avoid the decline of bone mass and bone quality. Second, the relationship between T1D and TBS was not affected by age. T1D most often appears in childhood and adolescence, with a high risk already present in childhood and continuing to increase throughout life span [11]. The association between T1D and decreased TBS found in this study in both adults and children not only indicates that trabecular bone is damaged by T1D in adults and children, but also indicates that decreased bone quality in T1D can occur before peak bone mass is reached. Therefore, in addition to insulin therapy, healthy lifestyle recommendations, including regular weight-bearing exercise, avoidance of smoking, adequate calcium intake, and vitamin D supplementation if necessary, are essential in the early stage of T1D, including children with T1D [54]. Finally, TBS was statistically associated with BMI and age; therefore, in the studies that corrected for age and/or BMI, we used the corrected mean. Furthermore, we performed subgroup analyzes with or without covariate adjustment and found that the association between T1D and TBS did not change regardless of age or weight adjustment.

This study has several limitations that should be noted. First, the small number of included studies and the small sample size may have adversely affected the interpretation of the results; Second, no study provided detailed TBS stratified by diabetes duration or sex, so we could not conduct subgroup analyses to explore different changes by diabetes duration and sex; Third, all the included studies were cross-sectional studies, and we could not effectively determine the causal relationship between T1D and TBS; Fourth, due to the lack of data, we cannot draw any conclusions about the causal relationship between low TBS and fracture occurrence; Fifth, different versions of the TBS software may also have induced bias in the study, but we used SMD as a measure of effect size, which may have controlled for differences in measurement.

In conclusion, this study showed that TBS was lower in patients with T1D than in healthy individuals with normal blood glucose levels, suggesting that TBS may be a useful measure for evaluating fracture risk in patients with T1D. Future prospective cohort studies with more representative population samples and more detailed data are needed to explore the relationship between T1D and TBS.

### Supplementary Information

**Additional file 1**. Supplementary Appendices for Tables and Material.

## Data Availability

All data included in this study are available upon request by contact with the corresponding author.
