# Supplementary material for: Trabecular bone score in type 1 diabetes: a meta-analysis of cross-sectional studies
Source: J Orthop Surg Res. 2023 Oct 24;18:794. doi: 10.1186/s13018-023-04289-0 (PMC10594696; doi:10.1186/s13018-023-04289-0)
Supplement: Supplementary file 1 — Additional file 1. Supplementary Appendices for Tables and Material. [file 13018_2023_4289_MOESM1_ESM.docx]

# Supplementary Appendices for Tables and Material

**PubMed Search Strategy**

((((trabecular bone score[Title/Abstract]) OR (TBS[Title/Abstract])) OR (osteoporosis[Title/Abstract])) OR (TBS[Title/Abstract])) OR (bone health[Title/Abstract])) AND (((((((Type 1 Diabetes[Title/Abstract]) OR (Insulin-Dependent Diabetes Mellitus[Title/Abstract])) OR (Juvenile-Onset Diabetes Mellitus[Title/Abstract])) OR (Sudden-Onset Diabetes Mellitus[Title/Abstract])) OR (Autoimmune Diabetes[Title/Abstract])) OR (Brittle Diabetes Mellitus[Title/Abstract])) OR (Ketosis-Prone Diabetes Mellitus[Title/Abstract]))

**EMBASE Search Strategy**

#1 AND #2

'type 1 diabetes':ab,ti OR 'insulin dependent diabetes mellitus':ab,ti OR 'insulin dependent diabetes mellitus':ab,ti OR 'insulin dependent diabetes mellitus':ab,ti OR 'insulin dependent diabetes mellitus':ab,ti OR 'brittle diabetes mellitus':ab,ti OR 'brittle diabetes mellitus':ab,ti

'trabecular bone score':ab,ti OR ' osteoporosis':ab,ti OR ' TBS':ab,ti OR ' bone health':ab,ti

**Web of Science Search Strategy**

#1 AND #2

((((((AB=(Type 1 Diabetes)) OR AB=(insulin dependent diabetes mellitus)) OR AB=(insulin dependent diabetes mellitus)) OR AB=(insulin dependent diabetes mellitus)) OR AB=(insulin dependent diabetes mellitus)) OR AB=(brittle diabetes mellitus)) OR AB=(brittle diabetes mellitus)

((AB=(trabecular bone score)) OR AB=(osteoporosis)) OR AB=(bone health) OR AB=(TBS)

| Author, Year | Selection | | | | Comparability | Outcome | | | Score |
| --- | --- | --- | --- | --- | --- | --- | --- | --- | --- |
|  | Representativeness | Selection of Non-Exposed | Exposure Ascertain | Outcome Absent | Comparability | Assess Outcome | Follow-Up Time | Follow-Up Adequacy |  |
| Shah VN, 2018 | * | * | * | * | ** | * | - | - | 7 |
| Syversen U, 2021 | * | * | * | * | * | * | - | * | 7 |
| Carvalho AL, 2018 | * | * | * | * | ** | * | - | - | 7 |
| Thangavelu T, 2020 | * | * | * | * | ** | * | - | - | 7 |
| Neumann T, 2015 | - | - | * | * | ** | * | - | - | 5 |
| Coll JC, 2022 | * | * | * | * | ** | * | - | - | 7 |
| Wagh A, 2021 | * | * | * | * | * | * | - |  | 6 |

Supplemental Table 1: Quality assessment of included studies using the Newcastle-Ottawa Scale
